# Adolescents’ Daily Race-Related Online Experiences and Mental Health Outcomes

**DOI:** 10.1001/jamanetworkopen.2025.36870

**Published:** 2025-10-07

**Authors:** Brendesha M. Tynes, Taylor McGee, Devin English

**Affiliations:** 1 University of Southern California, Los Angeles; 2 Christopher Newport University, Newport News, Virginia; 3 Rutgers School of Public Health, Newark, New Jersey

## Abstract

**Importance:**

Despite the rise in exposure to both positive and negative race-related experiences online, research on how these experiences are associated with mental health outcomes is limited.

**Objective:**

To examine the associations between negative online race-related experiences (online racial discrimination, traumatic events online, and algorithmic and filter bias), positive racial socialization messages, and mental health outcomes (depressive and anxiety symptoms).

**Design, Setting, and Participants:**

This survey study examined data from a nationally representative, intensive longitudinal 7-day daily dairy, collected as part of the National Survey of Critical Digital Literacy. Surveys were administered daily, with a 24-hour response window, in December 2020. Participants included Black or Black biracial or multiracial (parent-identified) adolescents. Participants were part of a larger nationally representative sample of 1138 adolescents, aged 11 to 19 years of various racial and ethnic backgrounds, recruited through Ipsos’ KnowledgePanel. One eligible adolescent per household was randomly selected to participate. Analyses were conducted in August 2021 and revised in July 2025.

**Main Outcomes and Measures:**

To examine the daily associations between online race-related experiences and mental health outcomes, dynamic structural equation models were estimated for the longitudinal data.

**Results:**

Results were drawn from survey responses of 141 Black and Black biracial or multiracial adolescents (mean [SD] age, 14.74 [2.51] years; 80 [56.7%] female). Adolescents reported a total of 6 online race-related experiences per day, including 3.2 that are considered online racism and 2.8 that are positive. The study found experiences of online racial discrimination (γ = 0.12; 95% credible interval [CrI], 0.01-0.21), algorithmic bias (γ = 0.11; 95% CrI, 0.02-0.19), and traumatic events online (γ = 0.10; 95% CrI, 0.01-0.19) were positively associated with next day anxiety symptoms. Online racial discrimination (γ = 0.20; 95% CrI, 0.01-0.33]), traumatic events online (γ = 0.10; 95% CI, 0.04-0.15), and algorithmic bias (γ = 0.13; 95% CrI, 0.03-0.22) were positively associated with next-day depressive symptoms. Positive racial socialization experiences were not associated with mental health outcomes.

**Conclusions and Relevance:**

In this survey study of online race-related experiences, Black adolescents, on average, reported experiencing 6 per day. Negative experiences were associated with poor mental health. Because young people can experience algorithms almost constantly, this study suggests more research is needed on related daily mental health outcomes.

## Key Points

**Question:**

What is the frequency of online race-related experiences and are positive and negative experiences online associated with mental health outcomes?

**Findings:**

Results of this longitudinal 7-day daily diary survey study including 141 adolescents showed that they reported a total of 6 online race-related experiences per day, including 3.2 that are considered online racism and 2.8 that are positive. Online racism was associated with poor next-day mental health outcomes.

**Meaning:**

These findings suggest adolescents report both online racism and positive race-related experiences, where they may learn about their racial or ethnic group’s survival despite difficult historical events; only online racism was associated with poor mental health.

## Teaser

This survey study evaluates the association between negative online race-related experiences, positive racial socialization messages, and mental health outcomes among US adolescents.

## Introduction

Though early scholarship suggested the internet could erase race and corresponding social ills, 2 decades of research show online race-related experiences have increased in importance and frequency.^1,2,3,4,5^ Studies have documented how young people express their racial identities, maintain friendships, and learn about within-group and outgroup experiences in online spaces.^1,6^ More recently, research has also shown that young people have frequent experiences with online racial discrimination and traumatic events online, including images or videos of racial terror in the form of police and state violence.^4,7^ These experiences are associated with depressive, anxiety, and posttraumatic stress symptoms, trauma symptoms of discrimination, and suicidal ideation.^3,4,7,8,9,10^ Despite the rise in exposure to both positive and negative race-related experiences online, including those powered by algorithmic racism, research on how these experiences are associated with mental health outcomes is limited.

Extensive use of the internet suggests a need for measures that are sensitive to daily life in digital spaces, particularly given that 56% of Black teens report being online “almost constantly” compared with 37% of White teens.^11^ The most common measurement approaches use large timeframes for assessment of discriminatory experiences (eg, 1 year)^12^ and increase the chance of retrospective bias,^13^ especially when considering nuanced microaggressions^14,15^ in an online setting. Moreover, this is particularly important among adolescents, who are in the process of major cognitive developments such as memory refinement, an essential mechanism for self-report measurement.^16^ However, studies that use daily diary methods can reduce retrospective biases inherent in self-report questionnaires, yielding data that are more reliable and accurate,^13^ thus providing a clearer picture of the frequency and outcomes associated with daily race-related experiences.

Given the potential of daily diaries, a number of studies have used this method to document associations between offline racial discrimination and psychological functioning.^17,18,19,20^ From these extant studies, including Douglass and colleagues’ 21-day daily diary study,^18^ we know that ethnic and racial teasing is associated with increased anxiety for those who are anxious. Similarly, a study from Cheeks et al^17^ included a 21-day daily diary of Black adolescents and showed discrimination is associated with negative psychological affect. To date, only 1 study in our review has examined both traditional and online daily racial discrimination. Among 101 Black adolescents, this study found that participants had a mean of 5.2 experiences per day, with those online being more frequent; these experiences were associated with depressive symptoms.^10^

Although comprehensive with respect to using both offline and online measures, previous research does not account for the range of race-related online experiences young people have, including both positive and negative experiences. Positive experiences may include educational material and conversations racial-ethnic groups of color may access to learn about themselves and their culture^1,6,21^ or negative experiences that may not have been captured in previous studies. For example, there have been many advances in technology, particularly with social media, that adolescents may experience daily, such as algorithmic bias.^22^

Our focus on algorithmic bias draws on recent research alternatively labeled “algorithmic oppression”^22^ or algorithmic racism, though with definitions that may differ slightly across studies or reports. We define algorithmic bias as a set of mathematical instructions that have centuries of racist policies and discrimination built into them^23^ and skew toward those in power. These instructions then drive automated decision-making. Scholars and journalists have noted different forms of algorithmic bias, such as a Google image search for “gorillas” yielding images of Black people or a search for the term “N**** House” leading to the White House when President Barack Obama was in office.^22,24^ To date, we know of no studies that have examined how adolescents’ experiences with algorithmic bias may be associated with mental health outcomes.

Similarly, it is unclear whether the algorithms of beauty filters, which augment digital images or videos in ways that often reinforce the superiority of Whiteness, negatively impact mental health. Research in this area focuses mostly on body image and is not race-related. For example, research suggests that Instagram, an image-based social media platform, is linked to social comparison, disordered eating, and poor body image.^25^ Studies also show that 48% of teens use beauty filters at least once each week, and 1 in 5 use them on every post.^26^ Sixty-one percent of these teens reported that beauty filters made them not like the way they look in real life. Given the widespread use of filters and their tendency to apply Eurocentric standards of beauty,^27^ more research accounting for these specific uses and their impact on mental health is needed.

Additionally, there is limited research on racial socialization via the internet.^28^ Scholars have demonstrated young people socialize with one another and share race-specific messages online.^1^ These messages may include positive messages about a person’s heritage or culture^6^ and educational sites such as the National Museum of African American History and Culture, which documents how Black people’s ingenuity, creativity, and labor have built and transformed US society. Moreover, while several studies have examined associations between negative race-related messages and mental health,^3,4,8,9^ it is unclear whether positive online messages impact adolescents’ mental health. This raises an empirical question; while one may not expect positive messages to negatively impact mental health, learning about the history and the challenges Black people have faced can be difficult. For example, traditional offline racial socialization messages that prepare young people for bias may be associated with increased depressive symptoms.^29^

We addressed noted gaps in the literature with the first comprehensive daily diary study of race-related experiences online that we know of, using a probability-based, nationally representative sample of Black adolescents. We also updated measures to account for recent developments in technology. Our measures of online racism, for example, include an item on deep fake videos and doctored images used to demean an individual because of their race. Additionally, we assessed a broader range of experiences that include race-related traumatic events online, algorithmic and filter bias, and positive racial socialization messages (PRS). We expected online racial discrimination, traumatic events online and algorithmic and filter bias to be positively associated with poor mental health outcomes, specifically depressive symptoms and anxiety. Due to the exploratory nature of the PRS, we did not have hypotheses for this variable.

## Methods

### Participants

Participants from the National Survey of Critical Digital Literacy (NSCDL) were recruited through Ipsos’ KnowledgePanel, the largest online panel in the US that relies on probability-based sampling methods for recruitment. Ipsos invited a parent or guardian from the representative sample of households with at least 1 eligible adolescent to enroll their adolescent(s) in the survey. Then, 1 adolescent per household was randomly selected to participate; adolescents participated after parental written consent and their own assent. Ipsos provided a nationally representative sample of 1138 adolescents, aged 11 to 19 years old. There was a target population of White, Black, Latine, and Black biracial or multiracial adolescents, with an oversampling of Black adolescents.

Five hundred and four participants were contacted from the full sample for the daily diary component of NSCDL. This study only focused on the responses of adolescents whose parent or guardian identified as Black and Black biracial or multiracial. Parents were contacted if they previously indicated they were White (non-Hispanic), Black or Black biracial or multiracial (non-Hispanic), or Hispanic for the daily diary component of the project. Note, wave 1 of the larger longitudinal mixed-methods study includes a self-report race and ethnicity question.

### Data Collection

This study was approved by the University of Southern California institutional review board. Data for this study were collected as part of the National Survey of Critical Digital Literacy which includes a longitudinal 2-wave mixed-methods component and an intensive longitudinal 7-day daily dairy. This study focused on data from the daily diary component.

Surveys were administered daily in the afternoon, with a 24-hour response window, from December 8 to 15, 2020. Measures assessed participants’ daily online digital literacy, race-related challenges and activities, and their depressive and anxiety symptoms. Participants took between 3 to 8 minutes to complete each day’s surveys. Participants completed a mean of 6 of 7 of the daily surveys, which is comparable with other daily diary studies with similar procedures.^10,30^ Standard email reminders were sent to nonresponders throughout the survey periods to encourage participation.

### Measures

#### Online Racial Discrimination

We used a revised version of the Online Victimization Scale to assess a range of adolescents’ experiences with individual online racial discrimination (ORD) (eg, “People have created deep fake (doctored) videos or photoshopped images of me to demean my race or ethnic group”).^31^ Participants were asked to think about their race-related experiences in the past 24 hours on a scale from 0 (did not happen) to 5 (happened 5 or more times).

#### Race-Related Traumatic Events Online 

Two items from the Race-Related Traumatic Events Online scale measured police and state racial terror (eg, I have seen a viral video of a Black person being killed by a police officer).^4^ Participants were asked to think about their race-related experiences in the past 24 hours on a scale from 0 (did not happen) to 5 (happened 5 or more times).

#### Algorithmic Bias

We created the Algorithmic and Filter Bias Scale^32^ for this study to assess bias and racism in algorithms young people encounter on a daily basis. Sample items include “I took a pic using a filter that made me look more European (e.g., lighter skin, straight hair)” and “I recognized that an algorithm produced biased results on a search I did.” Response options ranged from 0 (never) to 5 (constantly).

#### Positive Media and Online Racial Socialization

We created the Positive Media and Online Racial Socialization Scale^33^ for this study to measure participants’ daily online cultural socialization messages. Sample items included “In the past 24 hours, how many times did you…see a positive comment about your race or ethnic group on social media (e.g., TikTok, Twitter, Instagram, Snapchat, comments section, video games)? Or …see positive information about your race or ethnic group in educational or cultural websites (e.g., on a platform like Google Classroom and Schoology).” Response options ranged from 0 (did not happen) to 5 (happened 5 or more times).

#### Mental Health Outcomes

We used the Patient Health Questionnaire for Adolescents (a modified version of the PHQ-9), a multipurpose questionnaire for screening, diagnosing, and monitoring depression.^34^ We also used 2 items from the Generalized Anxiety Disorder-7 to assess anxiety symptoms.^35^ These questionnaires asked students to indicate how often they have been bothered by a list of problems (eg, trouble concentrating on things like school work, reading, or watching TV, and feeling nervous, anxious, or on edge). We revised the instructions to capture symptoms in the last 24 hours rather than the past 2 weeks. Response options ranged from 1 (not at all) to 4 (constantly).

### Statistical Analysis

To examine the daily associations between online race-related experiences and mental health outcomes, we estimated dynamic structural equation models (DSEM) for the intensive longitudinal data (ILD) produced by the daily surveys.^36^ DSEM is an analytic approach that combines time-series analysis, multilevel modeling, and structural equation modeling and models within-person lagged associations across many repeated measures, between-person differences in individual-level processes, and multiple latent and outcome variables.^37^ We followed best practices in 2-level DSEM for ILD in Mplus version 8.4 (Muthén and Muthén),^37^ with analyses as multilevel models that specify within-person variance (ie, change in outcome for a single person from day to day) and between-person variance (ie, differences between participants’ overall outcomes). The standard approach to DSEM within Mplus uses bayesian Markov Chain Monte Carlo estimation to aid in convergence and flexibility. In bayesian statistics, *P* values for effects are not produced, but typical frequentist inference can be approximated by examining whether a 0 is within the 95% credible interval (CrI) for each parameter. Intervals that contain 0 are analogous to not significant in a traditional frequentist analysis, while intervals that do not contain 0 are analogous to significant.

We ran separate sets of models to examine associations between each online race-related experience and mental health outcome separately (4 experiences × 2 outcomes = 8 models). In each model, we examined associations between experiences on day *t* − 1 and mental health outcome with a multilevel vector autoregressive models (VAR [1]) in the DSEM framework, which allows for multiple outcome variables on the within-state level.^36^ On the within level, the model included associations from experiences on day *t* − 1 to mental health outcomes on day *t* and adjusted for the reciprocal association from mental health outcomes on day *t* − 1 to experiences on day t, as well as autoregressive pathways from consecutive measurements of experiences and mental health outcomes. On the between-person level, the model included correlations between experience and mental health outcomes and associations between age and gender and both the experience and mental health outcome. DSEM notation and additional explanation is in the eMethods in Supplement 1.

Because we found a downward trend in depressive symptoms across the study period, such that depressive symptoms were negatively correlated with the previous day, we detrended the models by including time as a covariate. We estimated parameters via Markov Chain Monte Carlo with a Gibbs sampler using 2 chains thinned by 10 iterations with a minimum of 1000 iterations. We computed credible intervals with the highest posterior density method. We did not use restricted priors to estimate these models.

Regarding missing data, there was 100% complete data on race and ethnicity, age, and gender; 86% complete data on ORD, traumatic events online (TEO), anxiety, and depressive symptoms; and 85% complete data on algorithmic bias. This missingness was largely accounted for by missed survey days (86% completed surveys). The number of surveys completed was not significantly associated with age or gender of participants. To account for missing data, DSEM in Mplus uses a Kalman filter approach that incorporates predictions of missing observations using information from previous observations of that variable, allowing all observations to be incorporated into the analysis.^36^ Analyses were conducted in August 2021 and revised in July 2025.

## Results

Participants included 141 Black and Black biracial or multiracial adolescents (mean [SD] age, 14.74 [2.51] years; 80 [56.7%] female). For the ORD and TEO items, participants reported at least 2375 experiences of racial discrimination across 9 items during the 7-day daily period, including 1133 experiences across 7 ORD items and 1242 experiences across 2 TEO items. Participants experienced a mean (SD) of 0.32 (0.60) discrimination experiences per item administration, including 0.20 (0.52) ORD experiences and 0.75 (1.35) TEO experiences per administration, which translates to a mean of 2.9 experiences per day across 9 items, including 1.4 ORD across 7 ORD experiences and 1.5 across 2 TEO experiences. Thus, participants had a mean of 20.3 experiences per week. For the algorithmic bias items, because the scale did not measure frequency in the same approach as the ORD and TEO items, we report in terms of the mean score on the scale. Participants reported a mean (SD) per-day experience of 0.30 (0.61) which translates to having an experience 1 time per day every 3 days. Thus, participants reported a mean of 3.2 online racism experiences per day. For the PRS items, participants reported 2329 positive experiences across 4 items during the 7-day daily period. Participants experienced a mean (SD) of 0.70 (1.09) experiences per item administration, which translates to a mean of 2.8 experiences per day across 4 items. Thus, participants had a mean of 19.6 experiences per week.

For the DSEM models, there were no significant associations between time in the study and anxiety, indicating that we could assume stationarity among our outcomes for these models, though there was a time trend for depressive symptoms. Posterior distribution summaries, 95% CrIs, and within-person standardized effects are presented in the eTable in Supplement 1 for model intercepts and parameters of interest. For both depressive and anxiety symptoms across all models, there was significant between-person variability indicating the average of depressive and anxiety symptoms were different across people. Neither gender nor age were significantly associated with anxiety or depressive symptoms on the between-person level across all models.

For the ORD models, previous-day ORD experiences were positively associated with next day anxiety symptoms (γ = 0.19; 95% CrI, 0.01 to 0.38) and depressive symptoms (γ = 0.30; 95% CrI, 0.16 to 0.48). At the between-person level, ORD was positively correlated with anxiety (*r* = 0.07; 95% CrI, 0.03 to 0.14), indicating participants who experienced greater overall ORD also experienced higher anxiety, but ORD was not correlated with depressive symptoms (*r* = 0.01; 95% CrI, −0.02 to 0.06). Neither gender nor age were significantly associated with ORD in the anxiety model (gender: γ = −0.002; 95% CrI, −0.13 to 0.13; age: γ = −0.01; 95% CrI, −0.04 to 0.01) or depressive symptoms model (gender: γ = 0.04; 95% CrI, −0.07 to 0.16; age: γ = −0.01; 95% CrI, −0.04 to 0.02).

For the TEO models, previous-day TEO was positively associated with next day anxiety symptoms (γ = 0.06; 95% CrI, 0.01 to 0.11) and depressive symptoms (γ = 0.10; 95% CrI, 0.04 to 0.15). At the between-person level, TEO was positively correlated with anxiety, (*r* = 0.16; 95% CrI, 0.02 to 0.34), indicating participants who experienced greater overall TEO also experienced higher anxiety, but TEO was not correlated with depressive symptoms (*r* = 0.02; 95% CrI, −0.08 to 0.11). Neither gender nor age were significantly associated with TEO in the anxiety model (gender: γ = −0.02; 95% CrI, −0.36 to 0.32; age: γ = 0.04; 95% CrI, −0.02 to 0.11) or depressive symptoms model (gender: γ = 0.02; 95% CrI, −0.33 to 0.03; age: γ = 0.04; 95% CrI, −0.03 to 0.11).

For the algorithmic bias models, previous-day algorithmic bias was positively associated with next-day anxiety symptoms (γ = 0.16; 95% CrI, 0.01 to 0.29) and depressive symptoms (γ = 0.14; 95% CrI, 0.00 to 0.25). At the between-person level, algorithmic bias was positively correlated with anxiety (*r* = 0.05; 95% CrI, 0.01 to 0.11), indicating participants who experienced greater overall algorithmic bias also experienced higher anxiety, but algorithmic bias was not correlated with depressive symptoms (*r* = 0.01; 95% CrI, −0.03 to 0.05). Neither gender nor age were significantly associated with algorithmic bias in the anxiety model (gender: γ = 0.06; 95% CrI, −0.06 to 0.18; age: γ = −0.01; 95% CrI, −0.03 to 0.02) or depressive symptoms model (gender: γ = 0.08; 95% CrI, −0.04 to 0.20; age: γ = −0.004; 95% CrI, −0.03 to 0.02).

For the PRS models, previous-day PRS was not associated with next-day anxiety symptoms (γ = 0.06; 95% CrI, −0.03 to 0.13) nor depressive symptoms (γ = 0.06; 95% CrI, −0.002 to 0.14). At the between-person level, PRS was positively correlated with anxiety, (*r* = 0.11; 95% CrI, 0.01 to 0.22), indicating participants who experienced greater overall PRS also experienced higher anxiety, but PRS was not correlated with depressive symptoms (*r* = 0.01; 95% CrI, −0.07 to 0.10). Neither gender nor age were significantly associated with PRS in the anxiety model (gender: γ = −0.001; 95% CrI, −0.28 to 0.26; age: γ = −0.004; 95% CrI, −0.06 to 0.06) or depressive symptoms model (gender: γ = 0.06; 95% CrI, −0.22 to 0.27; age: γ = −0.001; 95% CrI, −0.07 to 0.06).

## Discussion

This survey study was the first we know of to use intensive longitudinal methods and a comprehensive measure to understand the association between positive and negative online race-related experiences and Black adolescents’ mental health. Hypotheses were confirmed for all online racism variables. Specifically, ORD, algorithmic bias, and TEO were all associated with increased next-day anxiety and depressive symptoms. Positive racial socialization experiences were not associated with poor mental health outcomes.

Participants reported experiencing both negative and positive experiences close to 3 times per day each. This is consistent with a previous daily diary study^10^ that showed Black adolescents reported 5.2 instances of racial discrimination per day with most of the experiences occurring online. Moreover, similar to this study,^10^ we found that experiencing racism online was associated with poor mental health the following day. This is also consistent with a study^38^ showing that over a 58-day study period, young people’s experiences of a single type of ORD (having people say mean or rude things about your ethnic group) was associated with same and next day’s poor mental health outcomes. Findings from the current study confirm this study grossly underestimated the number of adolescents experiencing ORD, however, with 1 of 2 noting they experienced at least 1 instance over the study period.

Perhaps most promising is that adolescents’ positive experiences with race online were not negatively associated with their mental health. Although they may encounter some negative historical and contemporary information about their culture or racial group, it does not have adverse consequences. This is consistent with cultural socialization research that suggests that learning about history and culture can be beneficial for young people’s well-being.^39^ Also, there is some literature on racial barrier messages that show no association with mental health; this literature is more equivocal, with some studies also suggesting a positive association and others a negative association.^39^ It should be noted that this study only assessed 4 types and contexts where adolescents experience positive messages. Future research should assess a wider range of messages and further explore the nature of these messages.

To our knowledge this is the first study to measure algorithmic bias using survey items among adolescents. It is also the first to determine the association with mental health. This is an important addition to the online racism literature, especially considering the rise in racism that is powered by racist algorithms.^40^ Scholars have discussed the potential harm algorithms may cause, but often without empirical data. Much of the research on the impact of algorithmic racism uses large datasets to calculate the harm on African Americans as a community or has noted the potential harms in search engines.^23^

### Limitations

This study has several limitations, including that data were collected during the COVID-19 pandemic when there was heightened internet use. Findings were consistent with previous daily diary studies of online racial discrimination collected prepandemic in terms of the frequency of negative race-related online experiences and mental health outcomes.^10^ Additionally, there were dramatic increases in the number of adolescents who said they were online “almost constantly” and “several times per day” from 2014 to 2018 due in part to the near ubiquity of smart phone use (95%) by 2018,^41^ well before the pandemic. Moreover, previously noted high percentages of Black teens who say they are online “almost constantly” were similar before and after the height of COVID-19.^11^ Taken together, previous findings and heightened internet use broadly suggest some consistency in adolescents’ digital experiences before and during data collection. Additional limitations include the binary, parent-reported gender variable used in this study; our broader study used a student measure with more options.

## Conclusions

In this intensive longitudinal daily diary study of race-related online experiences, we found ORD, TEO, and algorithmic bias were associated with next-day anxiety and depressive symptoms. Findings point to an urgent need for future research on young people’s race-related online experiences.

## Notes

Supplement 1. eMethods. Data Analytic Plan eTable. Dynamic Structural Equation Results eReferences

Supplement 2. Data Sharing Statement
