# Supplementary material for: Adolescents’ Daily Race-Related Online Experiences and Mental Health Outcomes
Source: JAMA Netw Open. 2025 Oct 7;8(10):e2536870. doi: 10.1001/jamanetworkopen.2025.36870 (PMC12505175; doi:10.1001/jamanetworkopen.2025.36870)
Supplement: Supplement 1. — eMethods. Data Analytic Plan eTable. Dynamic Structural Equation Results eReferences [file jamanetwopen-e2536870-s001.pdf]

Supplemental Online Content

Tynes B, McGee T, English D. Adolescents' daily race-related online experiences and mental health outcomes. *JAMA Netw Open*. 2025;8(10):e2536870. doi:0.1001/jamanetworkopen.2025.36870

eMethods. Data Analytic Plan

eTable. Dynamic Structural Equation Results

eReferences

This supplemental material has been provided by the authors to give readers additional information about their work.

© 2025 Tynes B et al. *JAMA Network Open*.

eMethods. Data Analytic Plan

As an example, the models examining the association between ORD and anxiety symptoms (Anx) can be written in statistically as:

Within-Person
$Anx_{ti} = \propto_{1i} + \varphi_{1i} Anx^{c}_{(t-1)i} + ß_{1i} ORD_{(t-1)i} + e_{1ti}$
$ORD_{ti} = \propto_{2i} + \varphi_{2} ORD^{c}_{(t-1)i} + ß_{2i} Anx_{(t-1)i} + e_{2ti}$
Between-Person
$\propto_{1i} = \gamma_{00+} u_{0i}$
$\varphi_{i} = \gamma_{10+} u_{1i}$
$ß_{i} = \gamma_{20+} u_{2i}$
$ORD^{b}_{i} = \gamma_{30} + Gen^{c}_{i} + Age^{c}_{i} + u_{3i}$
$Anx^{b}_{i} = \gamma_{40} + Gen^{c}_{i} + Age^{c}_{i} + u_{4i}$
The distributional assumptions were:
$e_{i} \sim N(0, \mathrm{diag}[\sigma_1^2, \sigma_2^2])$
$$u_i \sim N\left(0, \begin{bmatrix} \tau_{00} & & \\ \tau_{21} & \tau_{11} & \\ 0 & 0 & \tau_{22} \end{bmatrix}\right)$$
Where Anx is the anxiety symptoms outcome, *t* is time (in days, *t* = 1, …, 7), *i* is individuals (*i* = 1, … *N*), $\propto_{1i}$ is a person-specific intercept for anxiety, φ_i is the autoregressive effect measuring the carryover of anxiety symptoms from the previous day to the current day, Anx^c is latent-centered anxiety such that $Anx_i^c = Anx_i - \propto_{1i}$ which helps to address biases in time-series models,[1] $ß_{1i}$ is a time-varying covariate effect of ORD the previous day on anxiety symptoms during the current day, and e_{1ti} is a normally distributed within-person error with variance σ_1^2. The second expression in the within-person equation such that $\propto_{2i}$ is a person-specific intercept for ORD, φ_2 is the carryover effect of ORD on the current day from the previous day, and e_{2ti} is a normally distributed within-person error with variance σ_2^2.

In the between-person level, γ represents fixed effects that capture the average effect of the parameter across all persons, and *u* represents random effects that are normally distributed and include person-specific deviations from the fixed effects. Gen^c_i and Age^c_i are gender and age time-invariant covariates predicting ORD and anxiety between participants. The variances of the random effects are represented by the diagonal elements of the tau-matrix.

© 2025 Tynes B et al. *JAMA Network Open*.

eTable. Dynamic Structural Equation Results

Predictor	Outcome	Parameter	Est. (95% CI)[a]
ORD	Anxiety	Intercept, Fixed Effect	0.07 (-.64 to.67)
	Symptoms	Intercept, Variance	0.20 (.13-.31)
		ORD-Anx, Fixed Effect	0.19 (.01-.38)
		ORD-Anx, Variance	0.22 (.08-.40)
ORD	Depressive	Intercept, Fixed Effect	0.43 (-.06 to1.03)
	Symptoms	Intercept, Variance	0.12 (.08-.19)
		ORD-Dep, Fixed Effect	0.30 (.16-.48)
		ORD-Dep, Variance	0.21 (.10-.41)
TEO	Anxiety	Intercept, Fixed Effect	-0.11 (-.81to .47)
	Symptoms	Intercept, Variance	0.16 (.10-.24)
		TEO-Anx, Fixed Effect	0.06 (.01-.11)
		TEO-Anx, Variance	0.05 (.03-.08)
TEO	Depressive	Intercept, Fixed Effect	0.21 (-.30 to .71)
	Symptoms	Intercept, Variance	0.12 (.07-.18)
		TEO-Dep, Fixed Effect	0.10 (.04-.15)
		TEO-Dep, Variance	0.04 (.02-.07)
Algorithmic	Anxiety	Intercept, Fixed Effect	0.06 (-.50 to .63)
Bias	Symptoms	Intercept, Variance	0.14 (.09-.21)
		ALR-Anx, Fixed Effect	0.16 (.01-.29)
		ALR-Anx, Variance	0.29 (.17-.47)
Algorithmic	Depressive	Intercept, Fixed Effect	0.23 (-.34 to 75)
Bias	Symptoms	Intercept, Variance	0.12 (.08-.19)
		ALR-Dep, Fixed Effect	0.14 (.001-.25)
		ALR-Dep, Variance	0.12 (.06-.21)
Positive	Anxiety	Intercept, Fixed Effect	0.04 (-.41to .49)

© 2025 Tynes B et al. *JAMA Network Open*.

Experiences	Symptoms	Intercept, Variance	0.08 (.05-.13)
		POS-Anx, Fixed Effect	0.06 (-.03 to .13)
		POS-Anx, Variance	0.08 (.05-.18)
Positive Experiences	Depressive Symptoms	Intercept, Fixed Effect	0.25 (-.33 to .72)
		Intercept, Variance	0.11 (.07-.16)
		POS-Dep, Fixed Effect	0.06 (-.002 to.14)
		POS-Dep, Variance	0.11 (.07-.16)

Note: ORD = Online Racial Discrimination, TEO = Traumatic Events Online, ALR = Algorithmic Bias, POS = Positive Experiences, Anx = Anxiety, Dep = Depression, Est. = estimate or median of the posterior distribution, CI = Credible Interval using the highest posterior density method, [a] Statical significance in a frequentist inference occurs when 0 is not in the 95% credible interval of the parameter.

© 2025 Tynes B et al. *JAMA Network Open*.

eReferences

1. McNeish D, Hamaker EL. A primer on two-level dynamic structural equation models for intensive longitudinal data in Mplus. *Psychological Methods*. 2020; 25(5): 610-635. doi:10.1037/met0000250

© 2025 Tynes B et al. *JAMA Network Open*.
