# Supplementary material for: Adolescents’ Daily Race-Related Online Experiences and Mental Health Outcomes
Source: JAMA Netw Open. 2025 Oct 7;8(10):e2536870. doi: 10.1001/jamanetworkopen.2025.36870 (PMC12505175; doi:10.1001/jamanetworkopen.2025.36870)
Supplement: Supplement 2. — Data Sharing Statement [file jamanetwopen-e2536870-s002.pdf]

Data Sharing Statement

Tynes. Adolescents’ Daily Race-Related Online Experiences and Mental Health Outcomes. *JAMA Netw Open*. Published October 07, 2025. doi:10.1001/jamanetworkopen.2025.36870

Data
Data available: No
